# Body composition is a strong predictor of local carotid stiffness in Swedish, young adults – the cross sectional Lifestyle, biomarkers, and atherosclerosis study

**DOI:** 10.1186/s12872-019-1180-6

**Published:** 2019-08-27

**Authors:** Ulrika Fernberg, Jos op ‘t Roodt, Maria Fernström, Anita Hurtig-Wennlöf

**Affiliations:** 1 Cardiovascular Research Center, Faculty of Medicine and Health, Örebro University, Örebro, Sweden; 2 Department of Internal Medicine, Maastricht University Medical Center, Maastricht, The Netherlands; 3 CARIM, School for Cardiovascular Diseases, Maastricht, The Netherlands; 4 Åstrand Laboratory of Work Physiology, Swedish School of Sport and Health Sciences, GIH, Stockholm, Sweden; 5 School of Health Sciences, Örebro University, Fakultetsgatan 1, 701 82 Örebro, SE Sweden

**Keywords:** Arterial stiffness, Young adults, Body composition, Carotid artery, Arterial distensibility, Intima media thickness, Epidemiological, Cross-sectional study

## Abstract

**Background:**

Obesity has nearly tripled worldwide during the last four decades, especially in young adults, and is of growing concern since it is a risk factor for cardiovascular diseases (CVD). We explored how different body composition measurements are associated with intima media thickness (cIMT) and local stiffness in the common carotid artery, in a subsample of healthy, young women and men, from the Swedish Lifestyle, Biomarkers, and Atherosclerosis (LBA) Study.

**Methods:**

From the LBA study, a subsample of 220 randomly selected, self-reported healthy individuals, 18–25 years old, were collected for the automatized local stiffness measurements; arterial distensibility, Young’s elastic modulus, and β stiffness index. Blood pressure and mean arterial pressure (MAP) was measured using automatic blood pressure equipment. Body mass index (BMI) was calculated, waist circumference was measured, and percentage of body fat assessed using an impedance body composition analyzer. The carotid artery was scanned by ultrasound and analyzed using B-mode edge wall tracking. cIMT was measured and local stiffness measurements were calculated with carotid blood pressure, measured with applanation tonometry.

**Results:**

No association was found between cIMT and body composition. Local carotid stiffness was associated with body composition, and women had less stiff arteries than men (*p* < 0.001). Of the local stiffness measurements, arterial distensibility had the strongest associations with body composition measurements in both women and men (*p* < 0.05). Multiple regression analyses showed that BMI in women and BMI and percentage of body fat in men had the highest impact on arterial distensibility (*p* < 0.01 in both women and men).

**Conclusions:**

Arterial distensibility was the local stiffness measurement with the strongest associations to different body composition measurements, in both women and men. In this age group, body composition measurements seem to be stronger predictors of common carotid arterial stiffness than MAP, and is a convenient way of detecting young adults who need cardiovascular risk follow-up and lifestyle counseling.

## Background

The properties in the arterial tree which are of importance for cardiovascular health, do change over a lifetime [1]. There is a progressive stiffening of arteries along with healthy ageing, and this has been described in several longitudinal cohort studies [2–4]. The mechanical stress by the repetitive pulsations causes the elastin lamellae in the media to become frayed and fractured and the collagen fibers to increase. The elastic arteries respond with stiffening and dilation [5]. Arterial stiffness is associated with cardiovascular disease (CVD) risk factors and it is suggested that blood pressure and central body fatness plays an important role [4]. A systematic review indicates that obesity in children and adolescents is associated with greater arterial stiffness, as compared to healthy BMI controls [6]. A previous population-based study showed a positive association between local stiffness in the carotid and femoral arteries, all-cause mortality, and incidence of cardiovascular events [7]. An increase in carotid intima media thickness (cIMT) is also associated with many traditional risk factors and is considered to be a surrogate marker of atherosclerosis and increased risk of CVD [8]. Hypertension is suggested to be the risk factor that contributes most to an increase in cIMT, probably through medial hypertrophy [9].

Since the arterial properties is not uniform along the arterial tree it is important to measure arterial stiffness at different arterial sites [7]. Since atherosclerosis is common in the carotid artery, carotid stiffness can be of particular interest [10]. Increased carotid stiffness is associated with atherosclerotic plaque presence and stroke risk [11]. Carotid-femoral pulse wave velocity (cfPWV), which is a measurement of regional aortic stiffness, reflects the properties in a combination of elastic and muscular arteries. Local measurements from the carotid artery gives an understanding of stiffening in an elastic part of the arterial tree [7]. Therefore this present study will focus on local stiffness measurements and IMT in the elastic common carotid artery.

There are several descriptors of local carotid stiffness. The change in vessel diameter between systole and diastole is the absolute distention (systolic diameter (D_s_) – diastolic diameter (D_d_), μm). The distention is included together with local pulse pressure in the calculation of arterial distensibility (kPa^− 1^) [11]. The distensibility measures the ability of the arteries to expand in response to changes in blood pressure caused by cardiac relaxation and contraction. A formula that in addition to blood pressure also takes into account the arterial wall thickness, is Young’s elastic modulus (kPa). The cIMT is used as a surrogate for total arterial wall thickness in the Young’s elastic modulus formula [10]. Finally, β Stiffness index (unit-less), an index that accounts for the effect of blood pressure, by including the logarithm of the systolic to diastolic ratio in the equation, can be used to assess local arterial stiffness. The formulas for the local stiffness measurements are presented below [1]:

Arterial distensibility: (D_s_- D_d_) / ((Systolic pressure (P_s_) – Diastolic pressure (P_d_)) x D_d_).

Young’s elastic modulus: ((P_s_- P_d_) x D_d_)) / ((D_s_- D_d_) x h) were h is the arterial wall thickness.

β Stiffness index: (D_d_ ln(P_s_/P_d_)) / (D_s_- D_d_).

For interpretation, lower values of arterial distensibility and higher values of Young’s elastic modulus and β stiffness index, indicate stiffer vessels [11]. Because of pulse pressure amplification in young subjects, with a higher blood pressure in the peripheral arteries, it is of importance to use the local blood pressure from the same site as the relative diameter change is measured. The gold standard is to use the local blood pressure in the calculations of the different descriptors of local carotid elasticity [10]. The use of brachial pulse pressure may overestimate pulse pressure in central arteries, which results in false lower values of arterial distensibility and false higher values of Young’s elastic modulus and β stiffness index [12].

Obesity has nearly tripled worldwide during the last four decades and is of growing concern since it is a risk factor for CVD and several other non-communicable diseases [13]. According to WHO Body Mass Index (BMI) definitions [14], the Public Health Agency of Sweden reported in 2016 that 51% of the total Swedish population was overweight, and that overweight and obesity was increasing mostly in the age group between 16 and 29 years [15]. In addition to BMI, body composition can be assessed by measuring waist circumference, and the percentage of body fat can be assessed using an impedance body composition analyzer. Given the important role of arterial stiffness in CVD [7] and the worldwide increasing prevalence of overweight and obesity [13], it is important to find simple and useful methods for early identification of young adults with stiffening of the arterial tree, and increased CVD risk. It is of interest to explore how the body composition measurements (BMI, percentage of body fat, and waist circumference) are associated with local stiffness in the common carotid artery, measured by ultrasound and analyzed using B-mode edge wall tracking [16].

### Aim

The aim of the present study was to explore the hypothesis that local measurements of the common carotid artery are associated with body composition. The measurements used were thickness (cIMT) and stiffness, i.e., arterial distensibility, Young’s elastic modulus, and β stiffness index. The study was carried out in a subsample of healthy young women and men from the Swedish Lifestyle, Biomarkers, and Atherosclerosis (LBA) Study.

## Methods

### Study population

A highly specific vessel analysis of the common carotid artery [16] was performed on 220 individuals, randomly selected from the cross sectional LBA study (*n* = 834) [17]. The selected individuals were included in the subsample (here after called the LBA subsample) based on the Sphygmocor quality criteria and the ultrasound image. The quality criteria (pulse length variation, pulse height variation, shape deviation, and diastolic variation) in the Sphygmocor equipment needed to be fulfilled with as high quality index as possible (maximum 100%, no one had an index below 80%), and the near and far wall boundaries of the carotid artery needed to be clear and visible in the ultrasound image. The LBA subsample is representative of the LBA study with respect to gender distribution and also evenly distributed across the data collection period. The edge wall tracking of ultrasound B-mode recordings was not performed in all individuals in the LBA study due to technical and time limitations. The subsample was aiming to reach a fourth of the total LBA population. The individuals included in the LBA study were self-reported healthy without chronic disease, non-smoking, Swedish, young adults, 18–25 years old. All individuals gave their written consent to participate and the study was approved by the Regional Ethics Committee in Uppsala, Sweden (Dnr: 2014/224).

### Body composition

Height, weight, percentage of body fat, and waist circumference were measured with the subject in a fasting state. Height was measured to the nearest 0.5 cm with a fixed stadiometer. Waist circumference was measured with a measuring tape to the nearest 0.5 cm [18]. Weight was measured and percentage of body fat was calculated using an impedance body composition analyzer (Tanita Europe B.V. Tanita BC-418 MA, Amsterdam, Netherlands). BMI (kg/m^2^) was calculated and categorized into BMI ≤25 and BMI > 25.

### Biomarkers

Blood samples were collected with the individuals in a fasting state and venipuncture was performed after a resting period of approximately 20 min. Total cholesterol and glucose (mmol/L) were analyzed on an Ortho Clinical Diagnostics™ (Vitros 5.1TM FS; Clinical Chemistry Instruments, Raritan, NJ, U.S.A). Insulin (mU/L) was analyzed on an Architect i2000SR instrument from Abbott (Abbott Park, IL, USA). Analyses were performed at the accredited clinical chemistry laboratory at Örebro University Hospital. For more details, see the previously published description [17].

### Blood pressure and ultrasound measurements of the common carotid artery

**Fig. 1:**
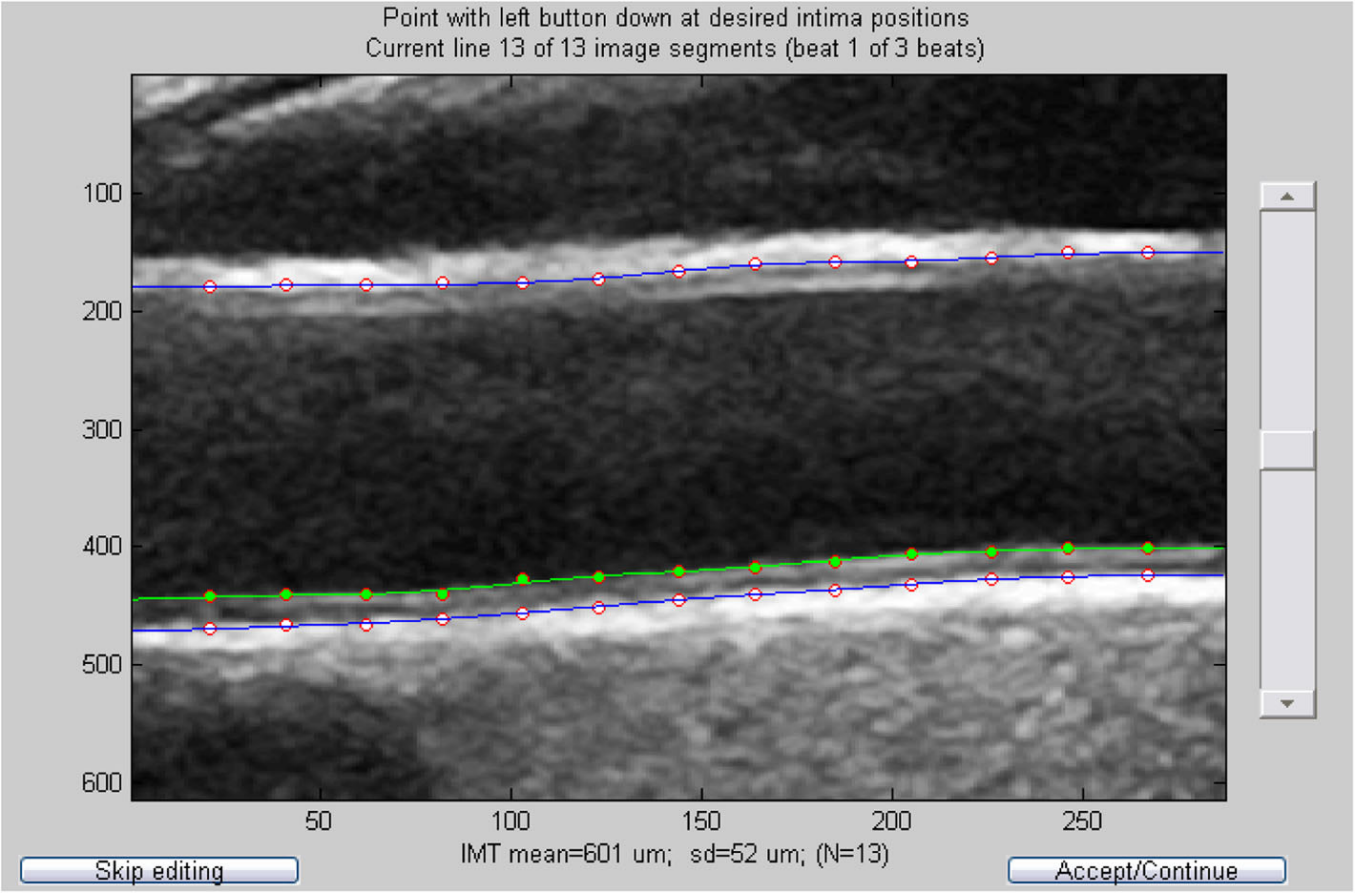
Automatic analysis of the common carotid artery distention and cIMT made with edge wall tracking of ultrasound B-mode recordings. The media-adventitia boundaries are defined by the blue lines and the distance between them is the carotid diameter. The lumen-intima boundary is shown as the green line and defines the cIMT on the far wall together with the blue line

Brachial blood pressure was measured with an oscillometric, non-invasive blood pressure method, after 10 min rest in a supine position, using a digital automated device (GE Healthcare, Dinamap V100, Buckinghamshire, UK) as earlier described [17]. Ultrasound measurements of the right common carotid artery were performed using a high-resolution B-mode system, (GE Healthcare, Vivid E9, Chicago, Illinois, US) with a 12 MHz linear array transducer. The subjects were examined in a supine position with their heads slightly extended and turned approximately 45 ^o^ to the left, according to guidelines [19, 20]. The right carotid artery was scanned with transverse and longitudinal views and a Doppler flow measurement was made to verify the location of the examination. A simultaneous ECG-recording was made during the ultrasound measurements. An automatic analysis of the common carotid artery distention and cIMT was performed with edge wall tracking of ultrasound B-mode recordings, see Fig. 1, using custom build Matlab software developed at Maastricht University Medical Centre (MUMC, Maastricht, The Netherlands) [16]. The software is based on previously published algorithms [21]. Analyses were performed by ultrasound specialist, blinded for the study. To assess the reproducibility of ultrasound edge wall tracking analyses, the coefficient of variation (CV) was calculated as ((standard deviation/mean) × 100). The CV for mean distention and mean cIMT were 6,0% and 6,9% respectively.

### Applanation tonometry of the common carotid artery

The right common carotid artery was also examined with applanation tonometry, SphygmoCor (AtCor Medical Pty Ltd., SphygmoCor, Sydney, Australia), in conjunction with a pulse wave velocity examination. Before the tonometry examination was performed, brachial blood pressure measurements were repeated to ensure a representative and stable blood pressure. The common carotid pulse waves were recorded with the subject in a supine position, in a temperature-controlled room (22-24 °C). At least three measurements were made on each test subject. The carotid blood pressure was obtained by a calibration method using the brachial artery pressure and wave, which is based on the observation, assuming that mean and diastolic blood pressure are constant throughout the large artery tree [22]. The measurement with the highest quality index on the SphygmoCor equipment [23] (pulse length variation, pulse height variation, diastolic variation and shape deviation) was reported for each subject.

### Calculations of local stiffness measurements

Calculations of the local stiffness measurements, arterial distensibility, Young’s elastic modulus, and β stiffness index were made according to the formulas presented in the background [1].

### Statistical analyses

All statistical calculations were made in females and males separately, and performed using IBM SPSS Statistics version 24 for Windows (IBM Corp, Armonk, New York, USA). Normal distribution was checked for all variables with Kolmogorov Smirnov test and Shapiro-Wilk test. Descriptive data are presented as mean and standard deviation. Unpaired Student’s *t*-test was used to analyze differences between the sexes and to analyze mean differences between the total LBA subsample (*n* = 220) and the LBA population (*n* = 834).

Simple linear regression were used to study associations between cIMT and the local stiffness measurements as dependent variables, and body composition measurements and blood pressure as independent variables, respectively. Correction for multiple comparisons were made according to Bonferroni. Based on the number of tests, the Bonferroni correction resulted in the following: Significant level < 0.05 requires *p* < 0.00104, significant level < 0.01 requires *p* < 0.00021, and significant level < 0.001 requires *p* < 0.000021. The local stiffness measure which had the strongest associations to the independent variables mentioned above (i.e. body composition measurements and blood pressure), was further explored in multiple regression analyses to assess the effect of the different body composition measurements on the dependent variable. The included variables, MAP and age, have earlier been described in the literature as determining factors for local stiffness measurements [1].

A regression model validation was made. Exclusion criteria’s were high values on Leverage and Cook’s distance, indicating outliers that could disturb the regression model, and high values on Variance inflation factors (VIF), indicating multicollinearity.

Unpaired Student’s *t*-test was used to study comparisons of mean values of arterial distensibility between the individuals with BMI ≤ 25 and the individuals with BMI > 25. Furthermore, the LBA subsample was split into the categories high body fat and low body fat by the median (gender specific) and an unpaired Student’s *t*-test was used to study comparisons of mean values of arterial distensibility between the individuals in the high body fat category and the low body fat category. The LBA subsample was also split into the categories high arterial distensibility and low arterial distensibility by the median (gender specific) and an unpaired Student’s *t*-test was used to study comparisons of mean values of percentage of body fat between the individuals in the high arterial distensibility category and the low arterial distensibility category.

## Results

### Study population

The LBA subsample (*n* = 220) did not differ in basic characteristics from the full LBA study population (*n* = 834) [17]. Basic characteristics and results of body composition measurements, blood pressure, carotid diameter, cIMT, and biomarkers are presented in women and men separately. In most variables, with exceptions for age, BMI, brachial diastolic blood pressure, and insulin there were significant differences between women and men, *p* < 0.05 (Table 1). There were significant differences between women and men (*p* < 0.001) in the calculated local stiffness measurements (Table 2). Women had higher arterial distensibility and lower Young’s elastic modulus, and β stiffness index than men, all *p* < 0.001.

**Table 1:** Body composition measurements, blood pressure, carotid diameter, cIMT, and biomarkers in the LBA subsample. Descriptives are presented for women and men separately

	LBA subsample (*n* = 220)						
	Women (*n* = 164)			Men (*n* = 56)			
	Mean	SD	Min-Max	Mean	SD	Min-Max	*P*-values
Age (year)	21.7	2.0	18–25	22.1	2.0	19–25	0.204
Height (cm)	168.3	6.1	149.0–189.0	181.1	6.0	169.0–200.0	< 0.001
Weight (kg)	62.6	10.7	42.8–139.0	74.8	9.9	57.3–96.1	< 0.001
BMI (kg/m^2^)	22.1	3.4	15.8–44.9	22.8	2.6	17.4–29.2	0.153
Waist (cm)	73.4	7.3	57.0–116.5	80.9	6.1	60.0–96.0	< 0.001
Body fat (%)	27.3	6.3	10.3–51.8	15.0	4.2	2.0–24.9	< 0.001
SBP_brach_ (mmHg)	110	8	90–138	123	10.0	103–148	< 0.001
DBP_brach_ (mmHg)	65	5	52–77	66	7	49–79	0.167
MAP_brach_ (mmHg)	81	6	40–98	86	7	68–99	< 0.001
Carotid diam (μm)	6028	352	5271–6843	6412	417	5592–7454	< 0.001
cIMT (μm)	610	81	400–819	646	105	453–901	< 0.05
Cholesterol (mmol/L)	4.3	0.8	2.5–8.2	4.0	0.8	2.5–5.8	< 0.01
Glucose (mmol/L)	4.9	0.3	4.2–6.9	5.2	0.3	4.5–5.9	< 0.001
Insulin (mU/L)	7.8	3.6	1.3–24.0	7.5	3.6	1.2–15.4	0.689

Notes: Results (mean, SD (standard deviation) and min-max values) are presented for women and men separately. Mean differences between the women and men were analyzed by unpaired Student’s t-test. Level of significance was set at *P < 0.05*

Abbreviations: *BMI (kg/m*^*2*^*)*, Body mass index, *Waist (cm)*, Waist circumference, *Body fat %*, percentage of body fat, *SBP*_*brach*_ *(mmHg)*, Brachial systolic blood pressure, *DBP*_*brach*_ *(mmHg)*, Brachial diastolic blood pressure, *MAP*_*brach*_ *(mmHg)*, Brachial mean arterial pressure, *Carotid diam (μm)*, Carotid end diastolic diameter, *cIMT (μm)*, Intima media thickness

**Table 2:** Results of local stiffness measurements in the common carotid artery, in the LBA subsample (n = 220)

	Women (*n* = 164)		Men (n = 56)		
Local stiffness variables calculated with carotid blood pressure	Mean	SD	Mean	SD	*P* values
Arterial distensibility (kPa^− 1^)	0.020	0.006	0.017	0.004	< 0.001
Young’s elastic modulus (kPa)	0.077	0.027	0.104	0.037	< 0.001
β Stiffness index	4.22	1.26	5.38	1.68	< 0.001

Notes: Results (mean and SD, standard deviation) are presented for women and men separately. Mean differences between the women and men were analyzed by unpaired Student’s t-test. Level of significance was set at *P < 0.05*

### Comparisons of local stiffness measurements

Simple linear regression models (Table 3) demonstrate significant inverse associations between arterial distensibility and BMI in both women and men (*p* < 0.01 and *p* < 0.05 in women and men, respectively). Significant inverse associations were also found between arterial distensibility and waist circumference in women (*p* < 0.05), and between arterial distensibility and percentage of body fat in men (*p* < 0.05). Among the blood pressure variables, significant inverse associations were seen between arterial distensibility and brachial systolic blood pressure in both women and men (*p* < 0.001 and *p* < 0.05 in women and men, respectively). Young’s elastic modulus was positively associated with BMI (*p* < 0.05) in women, but not in men. β stiffness index was also positively associated with BMI (*p* < 0.01) in women. No associations was seen between cIMT and the independent variables, (Table 3). Based on the observation that arterial distensibility was the local stiffness measurement that was significantly associated to most body composition measurements, in both women and men, the arterial distensibility was chosen as dependent variable in the multiple regression analyses to explore which of the body composition measurements, BMI, percentage of body fat, and waist circumference that contributed mostly to arterial distensibility.

**Table 3:** Simple linear regression with cIMT and local stiffness measurements as dependent variables

	cIMT (μm)		Arterial distensibility (kPa^−1^)		Young’s elastic modulus (kPa)		β stiffness index	
	Standardized β		Standardized β		Standardized β		Standardized β	
Variables	Women	Men	Women	Men	Women	Men	Women	Men
BMI (kg/m^2^)	0.147	0.172	− 0.310**	−0.437*	0.267*	0.325	0.294**	0.371
Waist circumference (cm)	0.138	0.150	−0.268*	− 0.390	0.233	0.245	0.250	0.254
Body fat (%)	0.178	0.199	−0.252	− 0.474*	0.214	0.255	0.239	0.323
SBP_brach_ (mm Hg)	0.242	0.146	−0.333***	−0.471*	0.241	0.299	0.154	0.247
DBP _brach_ (mm Hg)	0.164	−0.166	0.000	−0.082	−0.050	0.115	−0.201	− 0.099
MAP _brach_ (mm Hg)	0.204	−0.010	−0.170	− 0.272	0.155	0.262	0.015	0.092

Notes: Standardized β (Standardized coefficients β) are presented in women (*n* = 164) and men (*n* = 56) separately. Significance levels after Bonferroni correction; *P < 0.00104, **P < 0.00021, ***P < 0.000021

Abbreviations: *BMI (kg/m*^*2*^*)*, Body mass index, *Body fat (%)*, Percentage of body fat, *SBP*_*brach*_ *(mmHg)*, Brachial systolic blood pressure, *DBP*_*brach*_ *(mmHg)*, Brachial diastolic blood pressure, and *MAP*_*brach*_ *(mmHg)*, Brachial mean arterial pressure

### Associations between body composition measurements and arterial distensibility

Multiple regression analyses, with the variables known to affect arterial stiffness (age and MAP) [1] were performed with arterial distensibility as dependent variable and body composition measurements, BMI, percentage of body fat, and waist circumference as independent variables in separate models. In addition, the total cholesterol, glucose, and insulin, were also included in the models. The standardized β coefficients showed that BMI contributed slightly more to the variation in arterial distensibility than waist circumference and percentage of body fat in women (standardized β for BMI − 0.277, for waist circumference -0.218, and for body fat − 0.213). In men, the standardized β coefficients showed that BMI and percentage of body fat contributed equally to the variation in arterial distensibility (standardized β for BMI − 0.440, for body fat − 0.450, and for waist circumference − 0.375). Age, MAP, and the biomarkers contributed less than the body composition measurements to the variation in arterial distensibility in this age group (Table 4). Combining all three body composition measurements (BMI, percentage of body fat, and waist circumference) in a multiple regression model with arterial distensibility as dependent variable was not possible because of too high VIF values in the model validation, indicating multicollinearity between the independent determinants, as expected.

**Table 4:** Multiple regression models with arterial distensibility as dependent variable and BMI, percentage of body fat, and waist circumference as independent variables. The models are adjusted for age, MAP, and the biomarkers total cholesterol, glucose, and insulin

	Arterial distensibility (kPa^−1^)			
	Women (*n* = 164)		Men (*n* = 56)	
	Standardized β	P	Standardized β	P
BMI model				
BMI (kg/m^2^)	−0.277	< 0.01	−0.440	< 0.01
Age	0.013	0.874	−0.053	0.700
MAP_brach_ (mmHg)	−0.101	0.220	−0.177	0.183
Total cholesterol (mmol/L)	0.002	0.980	0.195	0.152
Glucose (mmol/L)	−0.064	0.449	−0.088	0.544
Insulin (mU/L)	0.048	0.617	0.028	0.843
Body fat model				
Body fat (%)	−0.213	< 0.05	−0.450	< 0.01
Age	−0.006	0.938	−0.159	0.208
MAP_brach_ (mmHg)	−0.113	0.174	−0.170	0.192
Total cholesterol (mmol/L)	0.015	0.856	0.199	0.133
Glucose (mmol/L)	−0.092	0.293	−0.109	0.440
Insulin (mU/L)	0.022	0.824	0.044	0.755
Waist model				
Waist (cm)	−0.218	< 0.05	−0.375	< 0.05
Age	−0.013	0.872	−0.096	0.488
MAP_brach_ (mmHg)	−0.115	0.165	−0.188	0.167
Total cholesterol (mmol/L)	−0.003	0.971	0.186	0.181
Glucose (mmol/L)	−0.068	0.432	−0.156	0.284
Insulin (mU/L)	0.019	0.845	0.082	0.600

Notes: Standardized β and *P* values for the independent variables are presented. Level of significance was set at *P < 0.05*

Abbreviations: *BMI (kg/m*^*2*^*)*, body mass index, *Waist (cm)*, waist circumference, *Body fat (%)*, percentage of body fat, *MAP*_*brach*_ *(mmHg)*, Brachial mean arterial pressure

**Fig. 2:**
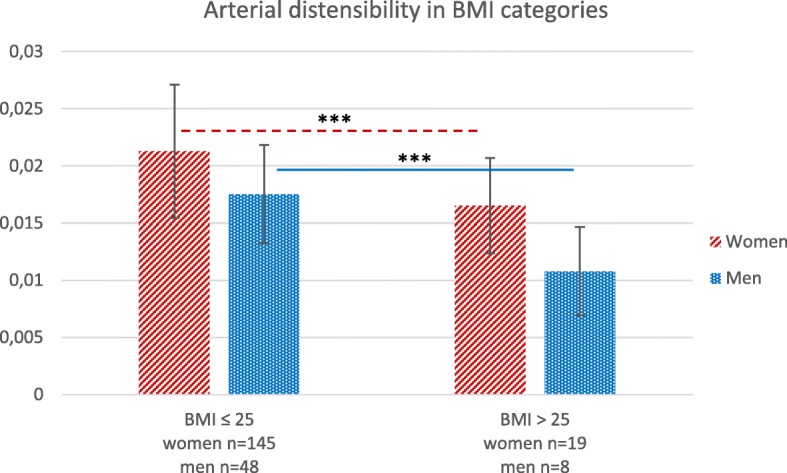
Arterial distensibility (kPa^− 1^) in the common carotid artery for women and men with BMI ≤ 25 and BMI > 25, ***P < 0.001. Low values indicate stiffer arteries. Arterial distensibility is calculated with carotid blood pressure. In women, four of 19 individuals with BMI > 25 were obese with a BMI > 30. In men, none of the eight individuals with BMI > 25 were obese. Whiskers represent +/− 1 standard deviation

There were significant differences in mean values of arterial distensibilty between the individuals with BMI ≤ 25 and the individuals with BMI > 25, both in women and men. Women and men with BMI > 25 had significantly lower arterial distensibility than the women and men with BMI ≤ 25 (*p* < 0.001 in both women and men), see Fig. 2. Furthermore, individuals in the high arterial distensibility group (i.e the individuals with more elastic arteries) had lower percentage of body fat. The individuals in the high body fat group had less elastic arteries (*p* < 0.05 and *p* < 0.01 respectively).

## Discussion

The main finding in the LBA subsample of healthy young individuals is that body composition measurements, especially BMI and percentage of body fat, are positively associated with a stiffer common carotid artery, in both young women and men.

### Methodological aspects

A decrease of arterial wall elasticity is a useful and early predictor for vascular disease. However, there is no precise direct method for the determination [24]. A lack of standards and the use of many different measures of local stiffness makes it difficult to compare different study results. Of the three local stiffness measurements that were calculated from the common carotid artery in the present study, arterial distensibility was the measure with the strongest associations with the body composition measurements, BMI, percentage of body fat, and waist circumference, in both women and men. The discrepancy between the three different local stiffness measurements is explained by the variables included in the calculations. When taking into account the intima media thickness that is included in the calculation of Young’s elastic modulus, associations with body composition measurements get weaker (in women) or disappear (in men). This could however be either a power issue because of the lower number of male individuals in the LBA subsample or the fact that the variation of cIMT in this young and healthy cohort was very low.

The guidelines recommend using the local blood pressure instead of the brachial blood pressure because of the pulse amplification between central and peripheral arterial sites, especially in young individuals [10]. In the present study, the local blood pressure in the common carotid artery was used in the calculations of the three local stiffness measurements, in opposite to several other studies using the brachial blood pressure [25–28]. One study reported that the brachial pulse pressure correlated well with the local pulse pressure both in women and men but the correlation was weakest in the youngest age group (r = 0.57) [28]. Since the study population in the present study is between 18 and 25 years, it is of importance to take into account that the stiffness measurements can be affected by the choice of blood pressure (e.g. brachial or carotid) used in the calculations.

### Comparisons of measurements in the common carotid artery between women and men

Significant differences were found between women and men in the common carotid artery diameter and cIMT, *p* < 0.001 and *p* < 0.05 respectively. Women had smaller carotid diameter (as expected) and thinner cIMT than men. In the Young Finns study, the cIMT differed between women and men in the same age group as the LBA subsample, also showing that women had thinner cIMT than men [29]. The same findings are demonstrated in a healthy sub-population in a multi-center study collecting reference intervals for carotid cIMT measured with echotracking [30].

Significant differences (p < 0.001) were also found between women and men in the calculated local stiffness measurements in the common carotid artery. Men had lower mean arterial distensibility and higher mean Young’s elastic modulus, and β stiffness index, indicating stiffer arteries than in women. The findings in the present study are in line with results from the Young Finns Study [27, 31]. They found that men had significantly lower carotid artery distensibility and higher pulse wave velocity, indicating stiffer arteries, compared to women in all age groups (30–36 years, 39–45 years, 46–76 years). The findings are also in line with the gender difference in pulse wave velocity, as a measurement of regional stiffness, which was demonstrated earlier in the LBA study, showing that men had higher pulse wave velocity than women [32].

Reference values for carotid distensibility coefficient, as a measurement of local arterial stiffness, were published in 2015 [28], in healthy test subjects, in the ages 15–85 years. The authors calculated carotid distensibility coefficient with artery area instead of diameter and found a negative and non-linear relationship between the carotid distensibility coefficient and age. When looking at the 50th percentile for the test subjects in the age of 20 years, the mean carotid distensibility coefficient was higher in women than in men, which is in line with the findings in the present study. Reference values from a younger age group, 6–18 years, have also been published, showing age- and sex-specific differences in distensibility measurements from the age of 15 years, were girls having higher mean value of carotid distensibility coefficient than boys [25]

### Local measurements of the common carotid artery and the influence of body composition

Previous data from the LBA study indicates that young, Swedish adults with obesity and low cardiorespiratory fitness have significantly higher cfPWV than non-obese adults with medium or high cardiorespiratory fitness [32]. The present study, with focus specifically on local stiffness in the common carotid artery, shows partly similar results. In the LBA subsample, women and men with BMI ≤ 25 and with lower percentage of body fat, had significantly higher arterial distensibility, indicating more elastic carotid arteries compared to women and men with BMI > 25 and a higher percentage of body fat. There are many instruments for the assessment of percentage of body fat, however there are no well-established cut-off values for the interpretation of the results.

Multiple regression analyses were performed to explore which of the body composition measurements that contributed mostly to the variation in arterial distensibility. The analyses showed that BMI had the highest impact on arterial distensibility in women, and that BMI and percentage of body fat had equal impact on arterial distensibility in men. One explanation to this gender difference could be the different fat distribution in women and men [33]. A man with a high percent of body fat is more likely to have a central/android fat distribution with fat stored preferentially in the abdominal area. In women it is more common with fat distributed to the hips and thighs [33]. Abdominal obesity, often assessed with waist-hip ratio, is associated with increased blood pressure, CVD, and diabetes [13, 34, 35]. This gender difference in fat distribution may explain why percentage of body fat have a greater impact on arterial distensibility in men, than in women.

### Strengths and limitations

There are several limitations in the present study, one is the lower number of male individuals. For unknown reasons it was much more difficult to recruit men to the study.

The possible number of edge wall tracking analyses were restricted by limited access to the wall tracking system and quality requirements of the registrations. A greater number of participants in the LBA subsample had been desirable to increase the power in the statistical calculations.

The fact that the individuals included in the study were self-reported healthy is another limitation in the study. We didn’t include individuals with a chronic disease but we accepted for example individuals with obesity, which is a reversible condition and an important risk factor for CVD.

Another limitation that needs to be highlighted is the cross-sectional design in the LBA study that prevents us from drawing conclusions in terms of causality.

One strength in the present study is the age of the population. Healthy young adults are underrepresented in the CVD literature compared to different patient groups. This age group can contribute to the detection of early changes affecting the development of CVD. The young adults in the age of 18–25 years are about to create their own habits and it is of great importance to highlight the benefits of a healthy lifestyle and to detect young adults who need cardiovascular risk follow-up and life-style counseling.

### Clinical perspectives

The body composition measurements, BMI, percentage of body fat, and waist circumference are common examinations that are easy to perform. In this age group, the body composition measurements contributed more than age, MAP, and the biomarkers to the variation in arterial distensibility. Our data suggests, that in this age group, BMI is the most simple and still useful tool to identify young adults that need to be further examined for future cardiovascular risk.

## Conclusions

Arterial distensibility was the local stiffness measurement with the strongest associations to the different body composition measurements, BMI, percentage of body fat, and waist circumference, in both women and men. In this age group, body composition seem to be a stronger predictor of common carotid arterial stiffness than MAP. BMI had the highest impact on arterial distensibility in women, and BMI and percentage of body fat had equal impact on arterial distensibility in men. Calculation of BMI is a convenient way of detecting young adults who need cardiovascular risk follow-up.

## Data Availability

All data generated and analysed during the current study are available from the corresponding author on reasonable request.

## Abbreviations

BMI: Body mass index
cfPWV: Carotid-femoral pulse wave velocity
cIMT: Carotid intima media thickness
CV: Coefficient of variation
CVD: Cardiovascular disease
DBP_brach_: Brachial diastolic blood pressure
LBA study: Lifestyle, biomarkers, and atherosclerosis study
MAP: Mean arterial pressure
MAP_brach_: Brachial mean arterial pressure
SBP_brach_: Brachial systolic blood pressure
Standardized β: Standardized coefficients β
VIF: Variance inflation factors

## References

[1] Nichols W, O'Rourke MF, Vlachopoulos C (2011). McDonald's blood flow in arteries.

[2] Mitchell GF, Parise H, Benjamin EJ, Larson MG, Keyes MJ, Vita JA (2004). Changes in arterial stiffness and wave reflection with advancing age in healthy men and women: the Framingham heart study. Hypertension.

[3] Gepner AD, Korcarz CE, Colangelo LA, Hom EK, Tattersall MC, Astor BC (2014). Longitudinal effects of a decade of aging on carotid artery stiffness: the multiethnic study of atherosclerosis. Stroke.

[4] Ferreira I, van de Laar RJ, Prins MH, Twisk JW, Stehouwer CD (2012). Carotid stiffness in young adults: a life-course analysis of its early determinants: the Amsterdam growth and health longitudinal study. Hypertension.

[5] O'Rourke MF, Hashimoto J (2007). Mechanical factors in arterial aging: a clinical perspective. J Am Coll Cardiol.

[6] Cote AT, Phillips AA, Harris KC, Sandor GG, Panagiotopoulos C, Devlin AM (2015). Obesity and arterial stiffness in children: systematic review and meta-analysis. Arterioscler Thromb Vasc Biol.

[7] van Sloten TT, Schram MT, van den Hurk K, Dekker JM, Nijpels G, Henry RM (2014). Local stiffness of the carotid and femoral artery is associated with incident cardiovascular events and all-cause mortality: the Hoorn study. J Am Coll Cardiol.

[8] Peters SA, Grobbee DE, Bots ML (2011). Carotid intima-media thickness: a suitable alternative for cardiovascular risk as outcome?. Eur J Cardiovasc Prev Rehabil.

[9] Pauletto P, Palatini P, Da Ros S, Pagliara V, Santipolo N, Baccillieri S (1999). Factors underlying the increase in carotid intima-media thickness in borderline hypertensives. Arterioscler Thromb Vasc Biol.

[10] Laurent S, Cockcroft J, Van Bortel L, Boutouyrie P, Giannattasio C, Hayoz D (2006). Expert consensus document on arterial stiffness: methodological issues and clinical applications. Eur Heart J.

[11] Boesen ME, Singh D, Menon BK, Frayne R (2015). A systematic literature review of the effect of carotid atherosclerosis on local vessel stiffness and elasticity. Atherosclerosis.

[12] Juonala M, Jarvisalo MJ, Maki-Torkko N, Kahonen M, Viikari JS, Raitakari OT (2005). Risk factors identified in childhood and decreased carotid artery elasticity in adulthood: the cardiovascular risk in young Finns study. Circulation.

[13] World Health Organization. Obesity and Overweight. https://www.who.int/en/news-room/fact-sheets/detail/obesity-and-overweight (cited 2017 December 15). (Webpage).

[14] World Health Organization. Body mass index - BMI. http://www.euro.who.int/en/health-topics/disease-prevention/nutrition/a-healthy-lifestyle/body-mass-index-bmi (cited 2019 August 08). (Webpage).

[15] Folkhälsomyndigheten (The Public Health Agency of Sweden). Övervikt och fetma. https://www.folkhalsomyndigheten.se/folkhalsorapportering-statistik/folkhalsans-utveckling/halsa/overvikt-och-fetma/ [cited 2017 December 15]. [Webpage].

[16] Steinbuch J (2017). In vivo ultrasound assessment of carotid artery walls and plaques.

[17] Fernstrom M, Fernberg U, Eliason G, Hurtig-Wennlof A (2017). Aerobic fitness is associated with low cardiovascular disease risk: the impact of lifestyle on early risk factors for atherosclerosis in young healthy Swedish individuals - the lifestyle, biomarker, and atherosclerosis study. Vasc Health Risk Manag.

[18] Lohamn T, Roche A, Martorell R (1988). Anthropometric standardization reference manual.

[19] Touboul PJ, Hennerici MG, Meairs S, Adams H, Amarenco P, Bornstein N (2012). Mannheim carotid intima-media thickness and plaque consensus (2004-2006-2011). An update on behalf of the advisory board of the 3rd, 4th and 5th watching the risk symposia, at the 13th, 15th and 20th European stroke conferences, Mannheim, Germany, 2004, Brussels, Belgium, 2006, and Hamburg, Germany, 2011. Cerebrovasc Dis.

[20] Stein JH, Korcarz CE, Hurst RT, Lonn E, Kendall CB, Mohler ER (2008). Use of carotid ultrasound to identify subclinical vascular disease and evaluate cardiovascular disease risk: a consensus statement from the American Society of Echocardiography carotid intima-media thickness task force. Endorsed by the Society for Vascular Medicine. J Am Soc Echocardiogr.

[21] Rossi AC, Brands PJ, Hoeks AP (2009). Nonlinear processing in B-mode ultrasound affects carotid diameter assessment. Ultrasound Med Biol.

[22] Kelly R, Fitchett D (1992). Noninvasive determination of aortic input impedance and external left ventricular power output: a validation and repeatability study of a new technique. J Am Coll Cardiol.

[23] Medical AC (2011). SphygmoCor research applications manual.

[24] Baltgaile G (2012). Arterial wall dynamics. Perspectives in Medicine.

[25] Doyon A, Kracht D, Bayazit AK, Deveci M, Duzova A, Krmar RT (2013). Carotid artery intima-media thickness and distensibility in children and adolescents: reference values and role of body dimensions. Hypertension.

[26] Jourdan C, Wuhl E, Litwin M, Fahr K, Trelewicz J, Jobs K (2005). Normative values for intima-media thickness and distensibility of large arteries in healthy adolescents. J Hypertens.

[27] Koskinen J, Magnussen CG, Viikari JS, Kahonen M, Laitinen T, Hutri-Kahonen N (2012). Effect of age, gender and cardiovascular risk factors on carotid distensibility during 6-year follow-up. The cardiovascular risk in young Finns study. Atherosclerosis.

[28] Engelen L, Bossuyt J, Ferreira I, van Bortel LM, Reesink KD, Segers P (2015). Reference values for local arterial stiffness. Part a: carotid artery. J Hypertens.

[29] Juonala M, Viikari JS, Kahonen M, Taittonen L, Laitinen T, Hutri-Kahonen N (2010). Life-time risk factors and progression of carotid atherosclerosis in young adults: the cardiovascular risk in young Finns study. Eur Heart J.

[30] Engelen L, Ferreira I, Stehouwer CD, Boutouyrie P, Laurent S (2013). Reference intervals for common carotid intima-media thickness measured with echotracking: relation with risk factors. Eur Heart J.

[31] Koivistoinen T, Virtanen M, Hutri-Kahonen N, Lehtimaki T, Jula A, Juonala M (2012). Arterial pulse wave velocity in relation to carotid intima-media thickness, brachial flow-mediated dilation and carotid artery distensibility: the cardiovascular risk in young Finns study and the health 2000 survey. Atherosclerosis.

[32] Fernberg U, Fernstrom M, Hurtig-Wennlof A (2017). Arterial stiffness is associated to cardiorespiratory fitness and body mass index in young Swedish adults: the lifestyle, biomarkers, and atherosclerosis study. Eur J Prev Cardiol.

[33] Karastergiou K, Smith SR, Greenberg AS, Fried SK (2012). Sex differences in human adipose tissues - the biology of pear shape. Biol Sex Differ.

[34] Yusuf S, Hawken S, Ounpuu S, Bautista L, Franzosi MG, Commerford P (2005). Obesity and the risk of myocardial infarction in 27,000 participants from 52 countries: a case-control study. Lancet..

[35] Canoy D, Luben R, Welch A, Bingham S, Wareham N, Day N (2004). Fat distribution, body mass index and blood pressure in 22,090 men and women in the Norfolk cohort of the European prospective investigation into Cancer and nutrition (EPIC-Norfolk) study. J Hypertens.

